# The Asylum Workers' Association

**Published:** 1912-05-11

**Authors:** G. E. Shuttleworth

**Affiliations:** Hon. Sec., Asylum Workers' Association. Parkholme, East Sheen, S.W.


*To the Editor of* The Hospital.

May 7.

### The Asylum Workers' Association.

*To the Editor of* The Hospital.

Sir,—I notice on page 116 of your last issue an annotation headed "Friction at a Mental Hospital," in which it is stated that the "local branch of the Asylum Workers' Association" has been making certain allegations with regard to the food supplied to the staff of the Cardiff Mental Hospital. As the Hon. Secretary of this Association, which was founded in 1895 with the object of "Improving the status of Asylum Nurses and Attendants and promoting their general welfare," may I state that no action has been taken by this Association, which has not a local branch at Cardiff, but it is probably due to the local branch of the "National Asylum Workers' Union," a trade-union organisation which was founded last year, the similarity of the name assumed being apt to give rise to confusion?—Yours faithfully,

G. E. Shuttleworth, Hon. Sec.,
Asylum Workers' Association.

Parkholme, East Sheen, S.W.,
May 7.

